# Diethyl 4,4′-{[1,2-phenyl­enebis(methyl­ene)]bis­(­oxy)}dibenzoate

**DOI:** 10.1107/S2414314625007898

**Published:** 2025-09-09

**Authors:** Sultana Shakila Khan, Md. Belayet Hossain Howlader, Ennio Zangrando, Md. Chanmiya Sheikh, Ryuta Miyatake

**Affiliations:** a Department of Pharmacy, Pabna University of Science and Technology, Pabna-6600, Bangladesh; b Department of Chemistry, Rajshahi University, Rajshahi-6205, Bangladesh; c Department of Chemical and Pharmaceutical Science, University of Trieste, Italy; d Division of Applied Chemistry, Graduate School of Natural Science, and Technology, Okayama University, 1-1 Tsushima-naka, 3-Chome, Okayama, 700-8530, Japan; e Center for Environmental Conservation and Research Safety University of Toyama, 3190 Gofuku Toyama 930-8555 Japan; Katholieke Universiteit Leuven, Belgium

**Keywords:** crystal structure, *ortho* disubstituted benzene, ester, ether

## Abstract

The title compound, C_26_H_26_O_6_, consists of two methyl­ene(­oxy)ethyl­benzoate moieties in *ortho* position to a phenyl ring. The central phenyl ring is almost coplanar with one of the ethyl­benzoate fragments, which guarantees an electron delocalization within the mol­ecule, while it forms a dihedral angle of 57.4 (2)° with the other. This conformation is likely dictated by packing requirements.

## Abstract 2

Two bulky methyl­ene(­oxy)ethyl­benzoate moieties located in *ortho* position to a phenyl ring.

## Graphical abstract

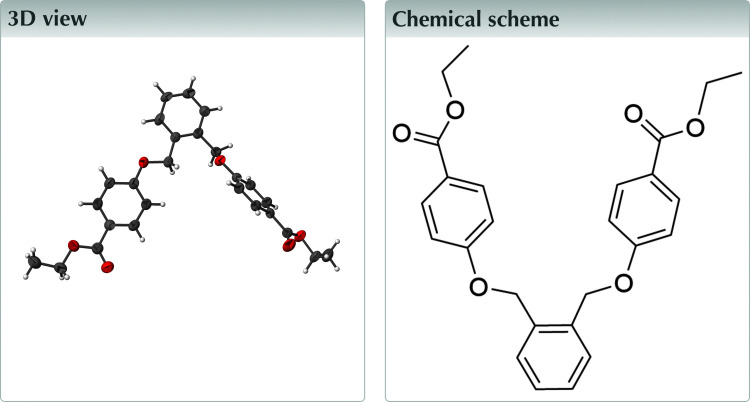

## Structure description

**Figure 1:**
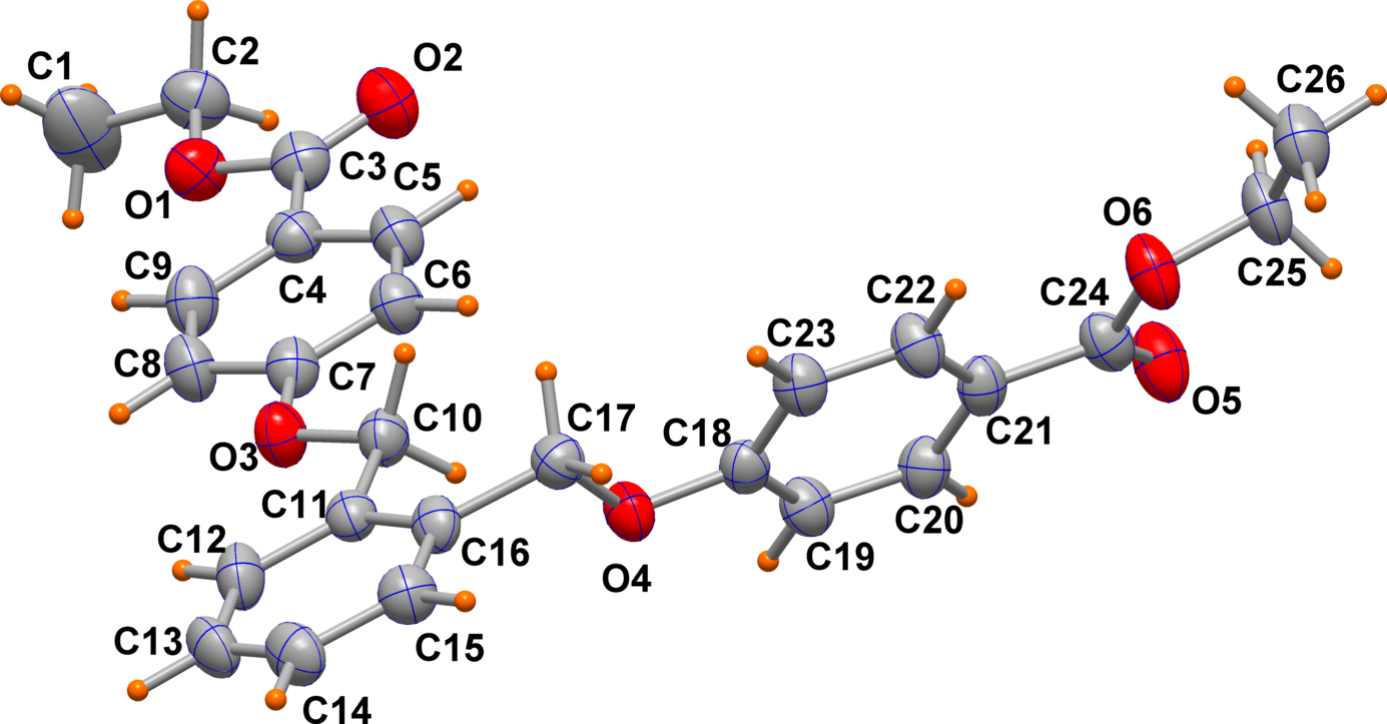
Mol­ecular structure of the title compound with ellipsoids drawn at the 50% probability level.

The title mol­ecule (Fig. 1 ▸) is formed by two methyl­ene(­oxy)ethyl­benzoate groups attached to adjacent (*ortho*) positions of a phenyl ring. The central C11–C16 phenyl ring has almost coplanar atoms with one of the methyl­ene(­oxy)ethyl­benzoate fragments (ring C4–C9), which ensures electron delocalization within the mol­ecule, while it forms a dihedral angle of 57.4 (2)° with the other ring (C18–C23). This conformation is likely dictated by crystal packing or in order to avoid intra­molecular steric clashes.

In the carboxyl­ate groups, the C3=O and C3—O bond lengths of 1.196 (5) 1.351 (5) Å are comparable to the C24=O and C24—O (in the second group) bonds of 1.208 (5), 1.343 (5) Å, respectively. All the geometrical parameters agree with those reported in similar species having a central ethane (Ma & Yang, 2011 ▸), propane (Li & Zheng, 2024 ▸) and octane chain (Khan *et al.*, 2022 ▸) replacing the benzene ring.

The mol­ecular structure is reinforced by C9—H9⋯O1, C12—H12⋯O3 and C22—H22⋯O6 short contacts [C⋯O distances of 2.746 (5), 2.714 (5) and 2.728 (5) Å, respectively].

**Figure 2:**
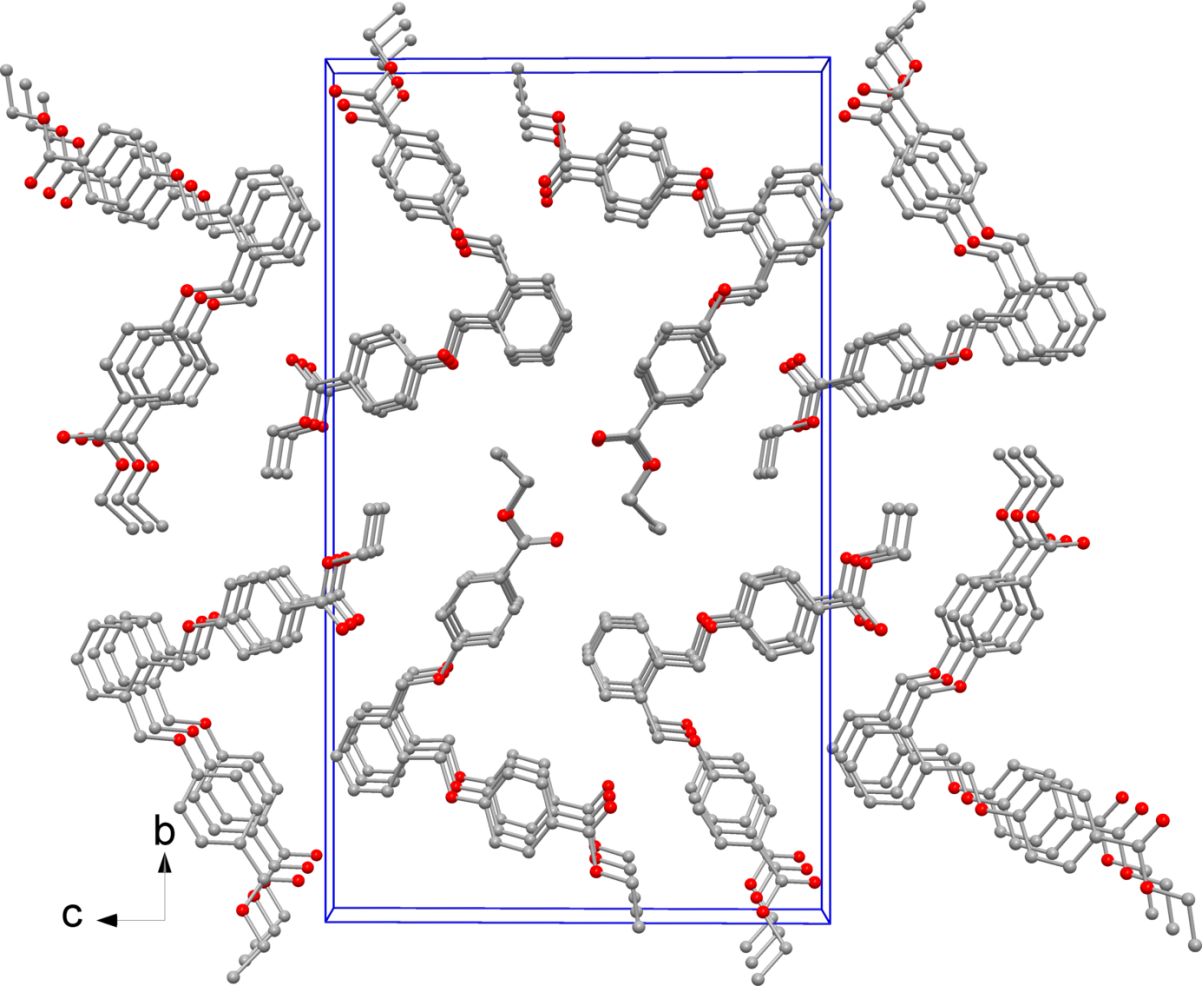
Detail of the crystal packing viewed down the *a* axis.

In the crystal packing (Fig. 2 ▸) the mol­ecules are stacked in the *a*-axis direction, favoring weak π-stacking inter­actions between C4–C9 phenyl rings [centroid-to-centroid distance of 4.109 (3) Å, but with a slippage of 2.134 Å]. Weaker C—H⋯O inter­actions are also detected among symmetry related mol­ecules in the crystal (Table 1 ▸).

For comparable mol­ecules bearing two methyl­ene(­oxy)ethyl­benzoate moieties, see Ma &Yang, 2011 ▸; Li & Zheng, 2024 ▸; Khan *et al.*, 2022 ▸. The corresponding species with *p*-oxy-benzoic groups was reported by Qiu *et al.* (2014 ▸), while the diethyl 2,2′-[1,3-phenyl­enebis(methyl­thio)]-dibenzoate was reported by Sillanpää *et al.* (1994 ▸).

## Synthesis and crystallization

A mixture of ethyl-4-hy­droxy­benzoate (8.35 g, 50 mmol) and αα^′^-di­bromo-*ortho*-xylene (6.63 g, 25 mmol) in acetone (100 ml) was refluxed for 24 h over anhydrous potassium carbonate (13.8 g, 100 mmol). The solvent was removed in a vacuum line, the solid mass was dissolved in water and extracted with di­chloro­methane and left overnight. A white precipitate was formed and filtered off. Colorless needle shaped crystals, suitable for X-ray diffraction, were formed after few weeks by slow evaporation from the solvent mixture of chloro­form, toluene and methanol (2:2:1, *v*/*v*/*v*), yield: 8.91 g (82%), melting point: 379–381 K.

FT–IR (KBr disc, cm^−1^): 3046 ν(C—H, aromatic), 1707 ν(C=O, ester) and 1603 ν(C=C, aromatic).

^1^H NMR (CDCl_3_, 400 MHz, p.p.m.), δ: 7.90 (*d*, 4H, C-5, 9, 20, 22, *J* = 8.8 Hz), 7.51 (*dd*, 2H, C-14, 15), 7.39 (*dd*, 2H, C-13,16), 6.97 (*d*, 4H, C-6, 8, 19, 23, *J* = 8.8 Hz), 5.22 (*s*, 4H, C-10, 17), 4.35 (*q*, 4H, C-2, 25), 1.37 (*t*, 6H, C-1, 26).

^13^C-NMR (CDCl_3_, 400 MHz, p.p.m.), δ: 166.36 (C-3, 24), 162.22 (C-4, 21), 134.61 (C-7, 18), 131.70 (C-5, 9, 20, 22), 129.27 (C-14, 15), 128.86 (C-13, 16), 122.50 (C-11, 12), 114.40 (C-6, 8, 19, 23), 68.20 (C-10, 17), 60.79 (C-2, 25), 14.46 (C-1, 26).

HRMS (FAB) Calculated for C_26_H_26_O_6_ [*M*+H]^+^: 435.18086, Found [*M*+H]^+^: 435.18022.

## Refinement

Crystal data, data collection and structure refinement details are summarized in Table 2 ▸.

## Supplementary Material

Crystal structure: contains datablock(s) I. DOI: 10.1107/S2414314625007898/vm4073sup1.cif

Structure factors: contains datablock(s) I. DOI: 10.1107/S2414314625007898/vm4073Isup2.hkl

Supporting information file. DOI: 10.1107/S2414314625007898/vm4073Isup3.cml

CCDC reference: 2335130

Additional supporting information:  crystallographic information; 3D view; checkCIF report

## Figures and Tables

**Table 1:** Hydrogen-bond geometry (Å, °)

*D*—H⋯*A*	*D*—H	H⋯*A*	*D*⋯*A*	*D*—H⋯*A*
C9—H9⋯O1	0.95	2.42	2.746 (5)	100
C12—H12⋯O3	0.95	2.35	2.714 (5)	102
C22—H22⋯O6	0.95	2.40	2.728 (5)	100
C17—H17*B*⋯O2^i^	0.99	2.54	3.429 (5)	150
C25—H25*A*⋯O5^ii^	0.99	2.60	3.234 (5)	122

Symmetry codes: (i) 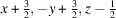; (ii) 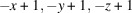.

**Table 2:** Experimental details

Crystal data	
Chemical formula	C_26_H_26_O_6_
*M*_r_	434.47
Crystal system, space group	Monoclinic, *P*2_1_/*n*
Temperature (K)	173
*a*, *b*, *c* (Å)	4.1087 (5), 30.414 (4), 17.782 (2)
β (°)	91.613 (7)
*V* (Å^3^)	2221.3 (5)
*Z*	4
Radiation type	Mo *K*α
μ (mm^−1^)	0.09
Crystal size (mm)	0.23 × 0.12 × 0.02

Data collection	
Diffractometer	Rigaku R-AXIS RAPID
Absorption correction	Multi-scan (*ABSCOR*; Higashi, 1995 ▸)
*T*_min_, *T*_max_	0.259, 0.998
No. of measured, independent and observed [*I* > 2σ(*I*)] reflections	18853, 4534, 2482
*R*_int_	0.116
(sin θ/λ)_max_ (Å^−1^)	0.625

Refinement	
*R*[*F*^2^ > 2σ(*F*^2^)], *wR*(*F*^2^), *S*	0.087, 0.218, 1.03
No. of reflections	4534
No. of parameters	291
H-atom treatment	H-atom parameters constrained
Δρ_max_, Δρ_min_ (e Å^−3^)	0.27, −0.31

Computer programs: *RAPID-AUTO* (Rigaku, 2010 ▸), *SHELXT2014/5* (Sheldrick, 2015*a* ▸), *SHELXL2019/2* (Sheldrick, 2015*b* ▸), *DIAMOND* (Brandenburg & Putz, 1999 ▸) and *WinGX* (Farrugia, 2012 ▸).

## full crystallographic data

### Diethyl 4,4'-{[1,2-phenylenebis(methylene)]bis(oxy)}dibenzoate Crystal data

**Table:**

C_26_H_26_O_6_	*F*(000) = 920
*M**_r_* = 434.47	*D*_x_ = 1.299 Mg m^−^^3^
Monoclinic, *P*2_1_/*n*	Mo *K*α radiation, λ = 0.71075 Å
*a* = 4.1087 (5) Å	Cell parameters from 8387 reflections
*b* = 30.414 (4) Å	θ = 1.8–27.4°
*c* = 17.782 (2) Å	µ = 0.09 mm^−^^1^
β = 91.613 (7)°	*T* = 173 K
*V* = 2221.3 (5) Å^3^	Platelet, colorless
*Z* = 4	0.23 × 0.12 × 0.02 mm

### Diethyl 4,4'-{[1,2-phenylenebis(methylene)]bis(oxy)}dibenzoate Data collection

**Table:**

Rigaku R-AXIS RAPID diffractometer	2482 reflections with *I* > 2σ(*I*)
Detector resolution: 10.000 pixels mm^-1^	*R*_int_ = 0.116
ω scans	θ_max_ = 26.4°, θ_min_ = 1.8°
Absorption correction: multi-scan (ABSCOR; Higashi, 1995)	*h* = −5→5
*T*_min_ = 0.259, *T*_max_ = 0.998	*k* = −38→38
18853 measured reflections	*l* = −22→19
4534 independent reflections	

### Diethyl 4,4'-{[1,2-phenylenebis(methylene)]bis(oxy)}dibenzoate Refinement

**Table:**

Refinement on *F*^2^	0 restraints
Least-squares matrix: full	Hydrogen site location: inferred from neighbouring sites
*R*[*F*^2^ > 2σ(*F*^2^)] = 0.087	H-atom parameters constrained
*wR*(*F*^2^) = 0.218	*w* = 1/[σ^2^(*F*_o_^2^) + (0.0571*P*)^2^ + 3.905*P*] where *P* = (*F*_o_^2^ + 2*F*_c_^2^)/3
*S* = 1.03	(Δ/σ)_max_ < 0.001
4534 reflections	Δρ_max_ = 0.27 e Å^−^^3^
291 parameters	Δρ_min_ = −0.31 e Å^−^^3^

### Diethyl 4,4'-{[1,2-phenylenebis(methylene)]bis(oxy)}dibenzoate Special details

**Table:**

Geometry. All esds (except the esd in the dihedral angle between two l.s. planes) are estimated using the full covariance matrix. The cell esds are taken into account individually in the estimation of esds in distances, angles and torsion angles; correlations between esds in cell parameters are only used when they are defined by crystal symmetry. An approximate (isotropic) treatment of cell esds is used for estimating esds involving l.s. planes.
Refinement. All H atoms were included at calculated positions and refined as riding atoms, with C–H = 0.90, 0.98, 0.99 \%A for aromatic, methylene and methyl groups, respectively, with *U*_iso_(H) = 1.2 *U*_eq_(C).

### Diethyl 4,4'-{[1,2-phenylenebis(methylene)]bis(oxy)}dibenzoate Fractional atomic coordinates and isotropic or equivalent isotropic displacement parameters (Å^2^)

**Table:**

	*x*	*y*	*z*	*U*_iso_*/*U*_eq_	
O1	−0.2918 (8)	0.92137 (9)	0.53618 (16)	0.0477 (8)	
O2	−0.3380 (8)	0.84948 (10)	0.56288 (18)	0.0553 (9)	
O3	0.4298 (7)	0.84798 (9)	0.25616 (16)	0.0442 (8)	
O4	0.8521 (7)	0.71712 (9)	0.22663 (15)	0.0407 (7)	
O5	0.8239 (9)	0.55812 (10)	0.45870 (16)	0.0558 (9)	
O6	0.5799 (8)	0.52849 (9)	0.35596 (16)	0.0505 (9)	
C1	−0.4652 (17)	0.97932 (17)	0.6157 (4)	0.087 (2)	
H1A	−0.601862	0.993692	0.576949	0.104*	
H1B	−0.241385	0.990233	0.612945	0.104*	
H1C	−0.549690	0.985915	0.665477	0.104*	
C2	−0.4690 (12)	0.93099 (15)	0.6034 (2)	0.0507 (12)	
H2A	−0.365891	0.915829	0.647167	0.061*	
H2B	−0.696252	0.920463	0.597415	0.061*	
C3	−0.2463 (11)	0.87813 (15)	0.5225 (2)	0.0430 (11)	
C4	−0.0620 (10)	0.87073 (14)	0.4522 (2)	0.0392 (10)	
C5	0.0038 (11)	0.82750 (14)	0.4326 (2)	0.0441 (11)	
H5	−0.066811	0.804081	0.463464	0.053*	
C6	0.1743 (11)	0.81840 (14)	0.3672 (2)	0.0423 (11)	
H6	0.224600	0.788910	0.354254	0.051*	
C7	0.2689 (11)	0.85272 (13)	0.3216 (2)	0.0396 (10)	
C8	0.2048 (12)	0.89609 (14)	0.3421 (2)	0.0451 (11)	
H8	0.275307	0.919702	0.311569	0.054*	
C9	0.0378 (12)	0.90450 (14)	0.4071 (2)	0.0457 (11)	
H9	−0.008234	0.934012	0.420670	0.055*	
C10	0.5033 (11)	0.80409 (13)	0.2329 (2)	0.0398 (10)	
H10A	0.299784	0.786924	0.226436	0.048*	
H10B	0.642730	0.789453	0.271740	0.048*	
C11	0.6795 (10)	0.80606 (13)	0.1589 (2)	0.0354 (10)	
C12	0.7625 (11)	0.84561 (13)	0.1265 (2)	0.0448 (11)	
H12	0.710554	0.872465	0.150633	0.054*	
C13	0.9232 (11)	0.84628 (14)	0.0580 (2)	0.0450 (11)	
H13	0.984284	0.873501	0.036313	0.054*	
C14	0.9919 (12)	0.80761 (15)	0.0224 (2)	0.0480 (12)	
H14	1.094006	0.808065	−0.024903	0.058*	
C15	0.9126 (11)	0.76768 (14)	0.0551 (2)	0.0423 (11)	
H15	0.966015	0.740945	0.030771	0.051*	
C16	0.7551 (10)	0.76661 (13)	0.1237 (2)	0.0373 (10)	
C17	0.6723 (10)	0.72256 (13)	0.1557 (2)	0.0379 (10)	
H17A	0.435428	0.720709	0.164025	0.045*	
H17B	0.731743	0.698988	0.120249	0.045*	
C18	0.8140 (10)	0.67807 (13)	0.2630 (2)	0.0356 (9)	
C19	0.9606 (11)	0.67506 (13)	0.3342 (2)	0.0409 (10)	
H19	1.076982	0.699462	0.354664	0.049*	
C20	0.9379 (11)	0.63689 (13)	0.3750 (2)	0.0401 (10)	
H20	1.042585	0.634982	0.423233	0.048*	
C21	0.7635 (10)	0.60098 (13)	0.3468 (2)	0.0371 (10)	
C22	0.6138 (11)	0.60482 (13)	0.2760 (2)	0.0425 (11)	
H22	0.492468	0.580709	0.256080	0.051*	
C23	0.6371 (11)	0.64284 (13)	0.2340 (2)	0.0401 (10)	
H23	0.532883	0.644826	0.185779	0.048*	
C24	0.7334 (11)	0.56101 (13)	0.3937 (2)	0.0409 (10)	
C25	0.4948 (13)	0.48973 (13)	0.3986 (2)	0.0482 (12)	
H25A	0.339269	0.497428	0.437999	0.058*	
H25B	0.691693	0.476743	0.423019	0.058*	
C26	0.3423 (13)	0.45777 (15)	0.3436 (3)	0.0589 (14)	
H26A	0.155045	0.471632	0.317818	0.071*	
H26B	0.269823	0.431611	0.370632	0.071*	
H26C	0.502635	0.449206	0.306554	0.071*	

### Diethyl 4,4'-{[1,2-phenylenebis(methylene)]bis(oxy)}dibenzoate Atomic displacement parameters (Å^2^)

**Table:**

	*U*^11^	*U*^22^	*U*^33^	*U*^12^	*U*^13^	*U*^23^
O1	0.052 (2)	0.0450 (18)	0.0462 (17)	0.0001 (15)	0.0076 (15)	−0.0061 (15)
O2	0.068 (2)	0.0485 (19)	0.0494 (19)	−0.0047 (17)	0.0078 (17)	0.0046 (16)
O3	0.057 (2)	0.0333 (15)	0.0428 (17)	0.0021 (14)	0.0062 (15)	−0.0005 (14)
O4	0.0489 (19)	0.0349 (15)	0.0381 (15)	−0.0042 (13)	−0.0019 (14)	0.0061 (13)
O5	0.087 (3)	0.0444 (18)	0.0361 (17)	−0.0071 (17)	−0.0070 (17)	0.0068 (15)
O6	0.080 (2)	0.0339 (16)	0.0376 (16)	−0.0082 (15)	−0.0001 (16)	0.0047 (13)
C1	0.106 (5)	0.064 (4)	0.094 (4)	0.007 (4)	0.053 (4)	−0.005 (3)
C2	0.052 (3)	0.057 (3)	0.043 (3)	−0.003 (2)	0.009 (2)	−0.004 (2)
C3	0.048 (3)	0.044 (3)	0.037 (2)	−0.002 (2)	−0.004 (2)	−0.002 (2)
C4	0.038 (2)	0.041 (2)	0.038 (2)	−0.0024 (19)	−0.0018 (19)	0.000 (2)
C5	0.052 (3)	0.036 (2)	0.044 (2)	−0.007 (2)	−0.001 (2)	0.005 (2)
C6	0.048 (3)	0.035 (2)	0.044 (2)	−0.001 (2)	0.001 (2)	−0.001 (2)
C7	0.047 (3)	0.035 (2)	0.036 (2)	0.0005 (19)	−0.005 (2)	−0.0017 (19)
C8	0.061 (3)	0.035 (2)	0.040 (2)	−0.002 (2)	0.000 (2)	0.005 (2)
C9	0.063 (3)	0.033 (2)	0.041 (2)	0.003 (2)	−0.002 (2)	−0.002 (2)
C10	0.044 (3)	0.032 (2)	0.044 (2)	0.0010 (19)	−0.001 (2)	0.0009 (19)
C11	0.035 (2)	0.035 (2)	0.036 (2)	−0.0021 (18)	−0.0052 (18)	0.0018 (18)
C12	0.059 (3)	0.030 (2)	0.045 (2)	−0.001 (2)	−0.010 (2)	0.004 (2)
C13	0.054 (3)	0.041 (2)	0.040 (2)	−0.010 (2)	−0.002 (2)	0.013 (2)
C14	0.056 (3)	0.053 (3)	0.035 (2)	−0.007 (2)	−0.001 (2)	0.010 (2)
C15	0.046 (3)	0.043 (2)	0.038 (2)	−0.001 (2)	0.000 (2)	−0.003 (2)
C16	0.043 (3)	0.034 (2)	0.034 (2)	−0.0009 (19)	−0.0085 (19)	0.0036 (18)
C17	0.040 (3)	0.039 (2)	0.034 (2)	−0.0006 (19)	−0.0002 (19)	0.0014 (19)
C18	0.039 (3)	0.034 (2)	0.034 (2)	0.0021 (18)	0.0059 (19)	0.0024 (18)
C19	0.048 (3)	0.034 (2)	0.041 (2)	−0.003 (2)	0.001 (2)	−0.004 (2)
C20	0.048 (3)	0.039 (2)	0.033 (2)	0.004 (2)	−0.001 (2)	0.0024 (19)
C21	0.046 (3)	0.031 (2)	0.034 (2)	0.0054 (19)	0.0058 (19)	−0.0012 (18)
C22	0.057 (3)	0.034 (2)	0.036 (2)	−0.005 (2)	0.000 (2)	−0.0010 (19)
C23	0.051 (3)	0.038 (2)	0.031 (2)	0.001 (2)	0.003 (2)	−0.0007 (19)
C24	0.050 (3)	0.035 (2)	0.039 (2)	−0.003 (2)	0.008 (2)	−0.001 (2)
C25	0.072 (3)	0.031 (2)	0.042 (2)	−0.006 (2)	0.000 (2)	0.008 (2)
C26	0.079 (4)	0.039 (3)	0.059 (3)	−0.004 (2)	−0.004 (3)	0.005 (2)

### Diethyl 4,4'-{[1,2-phenylenebis(methylene)]bis(oxy)}dibenzoate Geometric parameters (Å, º)

**Table:**

O1—C3	1.351 (5)	C11—C12	1.381 (6)
O1—C2	1.446 (5)	C11—C16	1.393 (5)
O2—C3	1.196 (5)	C12—C13	1.402 (6)
O3—C7	1.362 (5)	C12—H12	0.9500
O3—C10	1.432 (5)	C13—C14	1.369 (6)
O4—C18	1.364 (4)	C13—H13	0.9500
O4—C17	1.453 (4)	C14—C15	1.390 (6)
O5—C24	1.208 (5)	C14—H14	0.9500
O6—C24	1.343 (5)	C15—C16	1.396 (6)
O6—C25	1.449 (5)	C15—H15	0.9500
C1—C2	1.487 (7)	C16—C17	1.499 (5)
C1—H1A	0.9800	C17—H17A	0.9900
C1—H1B	0.9800	C17—H17B	0.9900
C1—H1C	0.9800	C18—C23	1.386 (5)
C2—H2A	0.9900	C18—C19	1.389 (6)
C2—H2B	0.9900	C19—C20	1.374 (5)
C3—C4	1.497 (6)	C19—H19	0.9500
C4—C9	1.373 (6)	C20—C21	1.392 (5)
C4—C5	1.389 (6)	C20—H20	0.9500
C5—C6	1.402 (6)	C21—C22	1.389 (5)
C5—H5	0.9500	C21—C24	1.482 (6)
C6—C7	1.384 (6)	C22—C23	1.381 (5)
C6—H6	0.9500	C22—H22	0.9500
C7—C8	1.395 (6)	C23—H23	0.9500
C8—C9	1.385 (6)	C25—C26	1.503 (6)
C8—H8	0.9500	C25—H25A	0.9900
C9—H9	0.9500	C25—H25B	0.9900
C10—C11	1.521 (6)	C26—H26A	0.9800
C10—H10A	0.9900	C26—H26B	0.9800
C10—H10B	0.9900	C26—H26C	0.9800
			
C3—O1—C2	114.9 (3)	C14—C13—H13	120.1
C7—O3—C10	117.2 (3)	C12—C13—H13	120.1
C18—O4—C17	116.7 (3)	C13—C14—C15	120.2 (4)
C24—O6—C25	117.1 (3)	C13—C14—H14	119.9
C2—C1—H1A	109.5	C15—C14—H14	119.9
C2—C1—H1B	109.5	C14—C15—C16	120.4 (4)
H1A—C1—H1B	109.5	C14—C15—H15	119.8
C2—C1—H1C	109.5	C16—C15—H15	119.8
H1A—C1—H1C	109.5	C11—C16—C15	119.2 (4)
H1B—C1—H1C	109.5	C11—C16—C17	122.9 (4)
O1—C2—C1	108.6 (4)	C15—C16—C17	117.9 (4)
O1—C2—H2A	110.0	O4—C17—C16	108.5 (3)
C1—C2—H2A	110.0	O4—C17—H17A	110.0
O1—C2—H2B	110.0	C16—C17—H17A	110.0
C1—C2—H2B	110.0	O4—C17—H17B	110.0
H2A—C2—H2B	108.4	C16—C17—H17B	110.0
O2—C3—O1	123.6 (4)	H17A—C17—H17B	108.4
O2—C3—C4	124.5 (4)	O4—C18—C23	124.2 (3)
O1—C3—C4	111.8 (4)	O4—C18—C19	115.9 (3)
C9—C4—C5	119.8 (4)	C23—C18—C19	119.8 (4)
C9—C4—C3	122.8 (4)	C20—C19—C18	120.2 (4)
C5—C4—C3	117.3 (4)	C20—C19—H19	119.9
C4—C5—C6	120.1 (4)	C18—C19—H19	119.9
C4—C5—H5	120.0	C19—C20—C21	120.9 (4)
C6—C5—H5	120.0	C19—C20—H20	119.5
C7—C6—C5	119.5 (4)	C21—C20—H20	119.5
C7—C6—H6	120.3	C22—C21—C20	118.1 (4)
C5—C6—H6	120.3	C22—C21—C24	122.5 (4)
O3—C7—C6	124.9 (4)	C20—C21—C24	119.4 (4)
O3—C7—C8	115.0 (4)	C23—C22—C21	121.6 (4)
C6—C7—C8	120.1 (4)	C23—C22—H22	119.2
C9—C8—C7	119.6 (4)	C21—C22—H22	119.2
C9—C8—H8	120.2	C22—C23—C18	119.3 (4)
C7—C8—H8	120.2	C22—C23—H23	120.4
C4—C9—C8	120.9 (4)	C18—C23—H23	120.4
C4—C9—H9	119.6	O5—C24—O6	123.5 (4)
C8—C9—H9	119.6	O5—C24—C21	124.8 (4)
O3—C10—C11	108.8 (3)	O6—C24—C21	111.6 (3)
O3—C10—H10A	109.9	O6—C25—C26	106.8 (3)
C11—C10—H10A	109.9	O6—C25—H25A	110.4
O3—C10—H10B	109.9	C26—C25—H25A	110.4
C11—C10—H10B	109.9	O6—C25—H25B	110.4
H10A—C10—H10B	108.3	C26—C25—H25B	110.4
C12—C11—C16	120.1 (4)	H25A—C25—H25B	108.6
C12—C11—C10	121.7 (4)	C25—C26—H26A	109.5
C16—C11—C10	118.2 (4)	C25—C26—H26B	109.5
C11—C12—C13	120.2 (4)	H26A—C26—H26B	109.5
C11—C12—H12	119.9	C25—C26—H26C	109.5
C13—C12—H12	119.9	H26A—C26—H26C	109.5
C14—C13—C12	119.9 (4)	H26B—C26—H26C	109.5
			
C3—O1—C2—C1	173.3 (4)	C10—C11—C16—C15	179.1 (4)
C2—O1—C3—O2	−1.4 (6)	C12—C11—C16—C17	180.0 (4)
C2—O1—C3—C4	179.9 (4)	C10—C11—C16—C17	−0.4 (6)
O2—C3—C4—C9	179.7 (4)	C14—C15—C16—C11	−0.3 (6)
O1—C3—C4—C9	−1.6 (6)	C14—C15—C16—C17	179.2 (4)
O2—C3—C4—C5	0.5 (6)	C18—O4—C17—C16	−178.1 (3)
O1—C3—C4—C5	179.2 (4)	C11—C16—C17—O4	−63.9 (5)
C9—C4—C5—C6	0.6 (6)	C15—C16—C17—O4	116.6 (4)
C3—C4—C5—C6	179.7 (4)	C17—O4—C18—C23	5.0 (6)
C4—C5—C6—C7	−1.6 (6)	C17—O4—C18—C19	−173.7 (4)
C10—O3—C7—C6	0.5 (6)	O4—C18—C19—C20	−179.5 (4)
C10—O3—C7—C8	179.6 (4)	C23—C18—C19—C20	1.7 (7)
C5—C6—C7—O3	−178.7 (4)	C18—C19—C20—C21	−1.2 (7)
C5—C6—C7—C8	2.3 (6)	C19—C20—C21—C22	0.1 (7)
O3—C7—C8—C9	179.0 (4)	C19—C20—C21—C24	−177.6 (4)
C6—C7—C8—C9	−1.9 (6)	C20—C21—C22—C23	0.5 (7)
C5—C4—C9—C8	−0.2 (7)	C24—C21—C22—C23	178.1 (4)
C3—C4—C9—C8	−179.3 (4)	C21—C22—C23—C18	0.0 (7)
C7—C8—C9—C4	0.8 (7)	O4—C18—C23—C22	−179.8 (4)
C7—O3—C10—C11	179.6 (3)	C19—C18—C23—C22	−1.1 (7)
O3—C10—C11—C12	3.7 (5)	C25—O6—C24—O5	5.8 (7)
O3—C10—C11—C16	−175.9 (3)	C25—O6—C24—C21	−171.7 (4)
C16—C11—C12—C13	0.0 (6)	C22—C21—C24—O5	−170.3 (5)
C10—C11—C12—C13	−179.6 (4)	C20—C21—C24—O5	7.3 (7)
C11—C12—C13—C14	1.4 (6)	C22—C21—C24—O6	7.1 (6)
C12—C13—C14—C15	−2.2 (7)	C20—C21—C24—O6	−175.2 (4)
C13—C14—C15—C16	1.6 (6)	C24—O6—C25—C26	−177.3 (4)
C12—C11—C16—C15	−0.5 (6)		

### Diethyl 4,4'-{[1,2-phenylenebis(methylene)]bis(oxy)}dibenzoate Hydrogen-bond geometry (Å, º)

**Table:**

*D*—H···*A*	*D*—H	H···*A*	*D*···*A*	*D*—H···*A*
C9—H9···O1	0.95	2.42	2.746 (5)	100
C12—H12···O3	0.95	2.35	2.714 (5)	102
C22—H22···O6	0.95	2.40	2.728 (5)	100
C17—H17*B*···O2^i^	0.99	2.54	3.429 (5)	150
C25—H25*A*···O5^ii^	0.99	2.60	3.234 (5)	122

Symmetry codes: (i) *x*+3/2, −*y*+3/2, *z*−1/2; (ii) −*x*+1, −*y*+1, −*z*+1.
